# Meta-models for rapid appraisal of the benefits of urban greening in the European context

**DOI:** 10.1016/j.ejrh.2021.100772

**Published:** 2021-04

**Authors:** Emanuele Quaranta, Chiara Dorati, Alberto Pistocchi

**Affiliations:** a European Commission, Joint Research Centre (JRC), Ispra, Italy; b ARHS Italia www.arhs-group.com, External Consultant for the European Commission, Italy

**Keywords:** Urban greening, Runoff, Energy, Urban heat island, Climate change mitigation

## Abstract

**Study region:**

This study considers daily time series of 14 years of weather parameters (temperature, wind speed, rainfall, vapor pressure and radiation) for 671 functional urban areas (FUA) across Europe, from a latitude of 35° (Cyprus) to 65° (Finland).

**Study focus:**

Quantification of urban greening effects usually requires relatively complex and integrated models. In this contribution, we apply well-established hydrological, biomass and energy balance equations to derive meta-models for the estimation of runoff reduction, urban surface heating and thermal protection of buildings, in order to quantify the effects of the greening of 1 m^2^ of impervious surface (e.g. roofs, sealed ground surfaces and underground parking lots).

**New hydrological insights for the region:**

We propose empirical meta-models for the quick appraisal of urban greening benefits including: urban runoff reduction due to soil water retention and evapotranspiration, land surface temperature reduction, reduction of the indoor temperature beneath the greened surface, dry biomass growth. We show that the choice of vegetation growth parameters has a limited effect on the results, although the amount of produced bulk biomass obviously depends on vegetation type. The proposed meta-models can be applied for the assessment of urban greening benefits at the stage of policy evaluation, land planning and the programming of investments at regional or continental scale, before undertaking more detailed and site-specific calculations as required in the design phase.

## Highlights

• A hydrological-energy-biomass model was applied to 1 m^2^ of green and grey surface.
• Biomass growth, surface temperature and runoff reduction due to the greening were calculated.
• Metamodels were derived to estimate greening benefits from climatic predictors.
• Metamodels can be used for regional-scale assessments for strategic planning.

## 1 Introduction

The resilience of cities under climate change is increasingly central in urban planning in Europe (European Commission, 2019) and worldwide, under sustained global urbanization trends (United Nations, 2019).

The impact of urbanization on climate has long been recognized (Chandler, 1962), and mitigation and adaptation strategies are extensively explored. Among these, urban greening has aroused considerable interest as a potential method to mitigate the effects of climate changes at the urban scale, particularly urban heat waves, droughts and floods. Plenty of studies have shown that the greening of grey surfaces through a vegetated cover on their top layer can deliver multiple benefits as they shift blue water (runoff) to green water through evapotranspiration, and store and gradually release rain water (Bowler et al., 2010; Chang et al., 2007; Spronken-Smith and Oke, 1998). Besides improving the urban habitat, with positive ecological and human well-being effects, greened surfaces may deliver at least the following benefits:

1 A mitigation of storm-water runoff by retaining precipitation, depending on the storage capacity allowed by the green surface and the local climatic pattern (Berndtsson, 2010; Schultz et al., 2018; Shafique et al., 2018; Soulis et al., 2017; Zhang et al., 2015). While surface greening alone has limited mitigating effects on extreme events, it can be effectively combined with other flood management measures (e.g. Mentens et al., 2006).
2 A mitigation of urban heat, as energy incoming to the surface is used for evapotranspiration instead of being absorbed (Issa et al., 2015; Susca et al., 2011). This reflects in a potential reduction of the energy needed for the cooling of buildings (La Roche and Berardi, 2014; Susca et al., 2011).
3 A fixation of atmospheric CO_2_ into biomass. If the biomass is properly managed, this could turn into carbon storage. Moreover, the biomass growing on greened surfaces may yield useful staple crops (Orsini et al., 2014).

Many studies have investigated the beneficial effects of urban greening (with a focus on green roofs) by means of field measurements. An accurate design of these complex systems usually requires rather detailed hydrological, thermodynamic and micrometeorological models that must be site-specifically applied (e.g. Locatelli et al., 2014; La Roche and Berardi, 2014; Issa et al., 2015). These are not practically applicable for the rapid appraisal of benefits at large scale, as required in early stages of programming and planning.

In order to overcome these difficulties, in this study we present simple equations able to surrogate more complex models with an acceptable approximation, for the general appraisal of policies and investments based on urban greening under varying climate conditions, representative of the European climate. Our equations are not conceived to accurately simulate local dynamics of greened surfaces, and only predict relatively simple and aggregated performance indicators of a greening intervention on an urban surface. Yet, their simplicity and immediateness of application make them suitable tools for screening-level studies. They enable quantitative cost-benefit appraisal that would not be possible in policy development and strategic planning, in the absence of resources to allocate to more complex modelling.

## 2 Materials and methods

The work consisted of two stages, namely:

(1) calculation of daily water and energy balances, and biomass growth, for a 1-m^2^ impervious area, initially without soil (grey surface), and then covered with soil and vegetation (green surface), using a combined hydrological, biomass growth and energy balance model;
(2) derivation of monovariate and multivariate regression equations to relate key output variables to appropriate predictors. These equations represent meta-models aimed at surrogating the underlying models in order to predict specific endpoints.

In this work, we do not restrict the definition of “urban greening”. The calculation applies to any surface initially paved with concrete, asphalt, tiles or similar materials, which is then covered with a layer of soil where vegetation is planted. Examples of surfaces that can be greened are roofs, sealed ground surfaces, or covers of underground parking lots. We expect that roof surfaces could be only covered with thin soil layers in the most common situations, while cases with less stringent structural constraints may warrant thicker soil covers. However, also on structurally constrained roofs it could be possible to implement discontinuous patches of thicker soils yielding an acceptable average thickness. For this reason, in the derivation of our meta-models we refer to a range of soil thickness beyond what is expected to be realistically applicable to green roofs alone.

### 2.1 Input data

The input climatic data were 14-years meteorological daily time-series (1990–2013) from the 5 × 5 km^2^ grid used in the hydrological model LISFLOOD (Bisselink et al., 2018). The daily grid values of the time series were averaged within the polygons representing 671 functional urban areas across Europe, spanning different geographic and climatic contexts at latitudes from 35° (Cyprus) to 65° (Finland), and from an average elevation of slightly below 0 m (−3 m, the Netherlands) to 1613 m (Innsbruck, Austria) (Pistocchi and Dorati, 2019). The climatic variables included in the daily time series were:

- precipitation *R* (mm),
- maximum and minimum temperature *T_max_* and *T_min_* (°C),
- vapor pressure *Vap* (hPa), wind speed *W* (m/s)
- daily average net short wave radiation *Rad* (kJ/m^2^).

From these data, the average temperature *T_av_* was calculated as the mean of maximum and minimum temperature. The long wave radiation was calculated by the equation proposed in Neitsch et al. (2011). The potential evapotranspiration *ET0* was calculated using the Penman-Monteith equation (see e.g. Neitsch et al., 2011) using the height of an herbaceous crop to compute the canopy aerodynamic resistance.

### 2.2 Combined hydrological, biomass and energy balance model applied at the daily scale

In the first stage, we applied the hydrological model proposed in Pistocchi et al. (2008), combined with the biomass growth and surface energy balance equations of the well-established Soil and Water Assessment Tool (SWAT) (Neitsch et al., 2011). The equations were solved in Matlab R2018a.

#### 2.2.1 Soil water balance

For a specific soil type and thickness, actual evapotranspiration (*AET*) was estimated as a function of potential evapotranspiration (*ET0*) and available water, neglecting vegetation canopy storage. This assumption is practical in order to avoid the need of an explicit water balance for the plants, including stem flow and evaporation from the leaves, which would arguably represent a higher-order refinement beyond the scope and needs of the present assessment. With this assumption, the hydrological and biomass models become independent of each other. The daily precipitation in excess of actual evapotranspiration is assumed to first replete the soil and then to originate runoff. The latter lumps together saturation and infiltration excess, as well as gravity drainage assumed to happen at the bottom of the soil layer. The soil water balance neglects all snow processes: in Europe, these are of relevance at high latitudes and altitudes and during winter, when vegetation growth is anyway severely limited by the temperature stress factor, and building cooling is not necessary. The impact of this simplification on the results of this analysis is therefore anticipated to be negligible.

#### 2.2.2 Biomass growth model

The soil water balance is computed using *ET0* and provides the soil water content used as an input to determine the water stress conditions possibly faced by plants. The potential biomass growth in kg/m^2^ was calculated at daily step using the equations of the SWAT model (Neitsch et al., 2011) from the intercepted photosynthetically active radiation (*PAR*), a function of atmospheric radiation, and radiation use efficiency (*RUE*) of the plant, a function of atmospheric vapor pressure. The actual biomass growth is then calculated through appropriate temperature and water stress factors depending on soil and weather conditions (Neitsch et al., 2011).

#### 2.2.3 Energy balance model

The net heat flux to the soil surface was calculated as the balance of net long wave radiation, net short wave radiation, actual evapotranspiration and heat flux through the soil. From this we computed the land surface (or skin) temperature under steady conditions for a 1 m^2^ surface with soil (*T_soil_*) and without soil, i.e. the grey surface (*T_grey_*). The surface temperature in the presence of vegetation *T_green_* was calculated from *T_soil_* and from the biomass weight load, in order to account for the influence of plant canopy on soil temperature (Neitsch et al., 2011). The summer average temperature values where then computed. Finally, the temperature at the bottom of the soil layer (*T_base_*) was estimated after taking into account the thermal inertia of the soil, depending on soil thickness *t* and soil water content (Nietsch et al., 2011). The difference Δ*T_ext_* = *T_grey_ – T_green_* between the surface temperature of the green surface completely covered by vegetation and the external grey surface temperature is an indicator to quantify the improvement of urban microclimate. The difference Δ*T_int_* = *T_grey_* – *T_base_* is instead an indicator of the change in temperature at the outer layer (external roof surface) of a building, and is proportional to the reduction of cooling energy requirements in summer if we assume that the building internal volume under the roof is to be cooled.

### 2.3 Choice of soil and vegetation parameters for input to the model and thermal boundary conditions

As surface greening requires importing soil specifically gathered for the purpose, a medium texture soil is usually to be preferred for agronomic reasons. For this reason we referred our calculations to a loamy soil corresponding tothe “medium” textural class as per Wösten et al. (1999) (see Table 2 in Pistocchi et al., 2008, for the hydrological properties of such soil). We considered five soil thicknesses (*t* =0.05 m, *t* =0.10 m, *t* =0.20 m, *t* =0.30 m and *t* =0.50 m) in order to obtain results potentially applicable in different contexts. We would like to stress once more that, in many cases, the soil thickness applicable for the greening of surfaces is limited due to structural or architectural constraints. In other cases, a thicker layer may be warranted. In order to cover the full range of solutions that could be implemented at least in some circumstance (including the greening of parts of sealed lots such as parking spaces), we developed our calculations for soil thicknesses up to 50 cm. Furthermore, it is reasonable to think that thicker, discontinuous soil patches could be also used on roofs, while maintaining a compatible average thickness.

Aboveground biomass growth was simulated using the SWAT model equations. We referred to a herbaceous plant, representing grassed surfaces, with plant growth parameters for *Medicago sativa* (alfalfa) suggested in SWAT (Neitsch et al., 2011). These parameters can be regarded as representative of grass in general, but affect the final results to a marginal extent, as the dry matter potentially produced per unit surface is relatively independent of the herbaceous plant (Li et al., 2014). A sensitivity analysis is presented in Appendix A to further corroborate this observation. The choice of plants to use in the design of urban greening depends anyway on a number of factors not considered here. When the plant reaches maturity, we assume it is cut down to the height of 3 cm, which is the minimum height suggested from the SWAT.

The flow of heat through the soil layer is only relevant to compute indicators of potential energy saving in the cooling of buildings. In our exercise, it was computed with reference to the scheme of a green roof covering a building with an internal temperature *T_int_* = 20 °C and a transmittance of the roof structure underlying the soil equal to 0.30 W m^−2^ K^-1^. The latter can be regarded as a typical European value (European Insulation Manufacturers Associations, 2017). Anyway, the results related to temperature differences do not significantly change by changing the inner building temperature *T_int_*.

### 2.4 Definition of performance indicators and development of meta-models

For each of the 671 functional urban areas, we computed the following performance indicators, for the different soil thicknesses and vegetation types, with reference to a 1 m^2^ urban area undergoing greening:

- the average yearly % reduction of runoff RR, assuming runoff for the urban surface prior to greening equals the average yearly rainfall, R_av,y_.
- Difference in ground surface temperature ΔT_ext_, expressed in °C and averaged over summer months, between the soil vegetated surface T_green_ and the surface prior to greening T_grey_.
- Dry biomass production CB, espressed in kg m^−2^ y^-1^.
- An indicator of the potential benefit for the cooling of buildings. This was defined as the difference in temperature at the outer skin of the roof beneath the soil layer, ΔT_int_, expressed in °C and averaged over summer months, between the temperature computed for a grey surface T_grey_ and the temperature at the bottom of the soil T_base_ in case of greening. The indicator ΔT_int_ is proportional to the change in heat flow from the outside to the inside of a building through a soil-covered roof, hence to the change in energy required for cooling if we assume that the building internal volume under the roof is to be cooled.

**Fig. 1:**
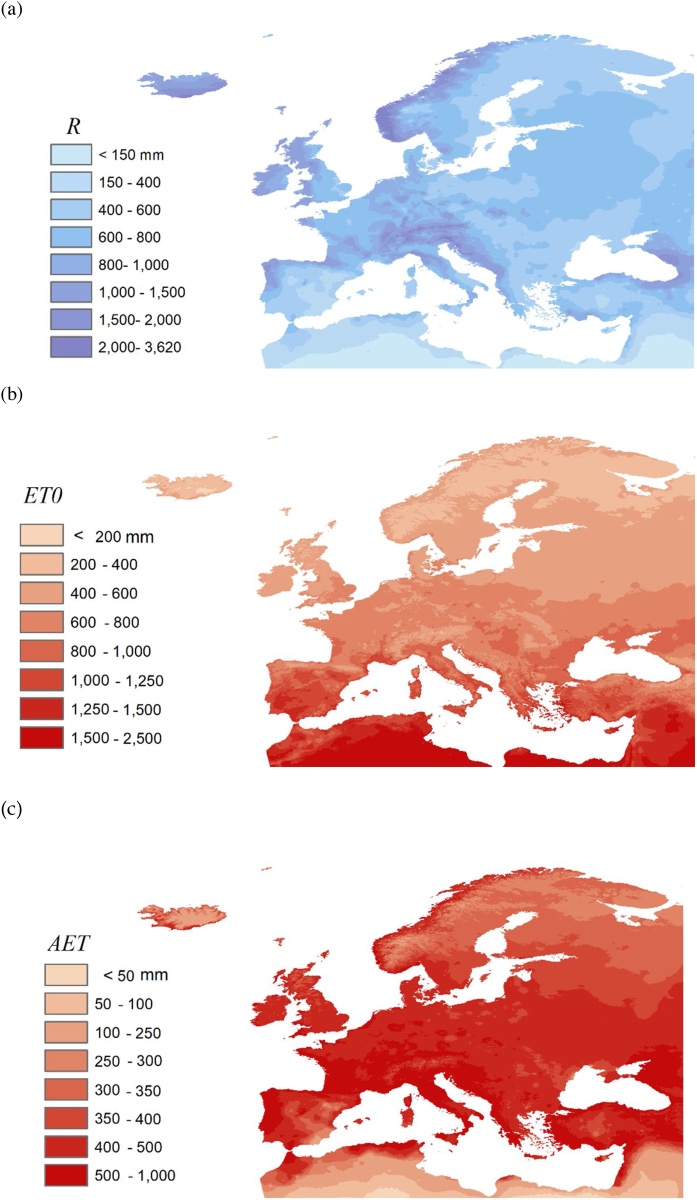
Maps of climatic predictors for each FUA: (a) Precipitation *R_av,y_*, (b) potential evapotranspiration *ET0_av,y_* and (c) actual evapotranspiration *AET_av,y_*.

For each of the above indicators, we developed regression equations using weather forcing variables as predictors. In particular we considered the annual average of the following variables (Fig. 1):

- yearly average precipitation R_av,y_ (mm), i.e. cumulated value in one average year, that ranged between 200 and 1600 mm (Fig. 1a);
- average daily short wave radiation (Rad, W/m^2^), that ranged between 100 and 240 W/m^2^;
- yearly average temperature (T_med_, °C), ranging between 3 and 20 °C;
- yearly average potential evapotranspiration (ET0_av,y_, mm), i.e. cumulated value in one average year, that ranged between 400 and 1400 mm (Fig. 1b); the humidity index HI = R _av,y_ / ET0_av,y_ ranges between 0.24 and 2.64.
- yearly average actual evapotranspiration (AET _av,y_, mm), computed by averaging the daily values obtained with the hydrological model mentioned above, and ranging between 150 and 550 mm for a soil 0.30 m thick and with herbaceous plants (Fig. 1c).
- average actual evapotranspiration during summer months (AET _av,s_, mm), ranging between 20 and 250 mm;

The above predictors can be easily obtained from weather observations, with the exception of actual evapotranspiration. The latter derives from a water balance and entails a more complex calculation. Its long-term annual average, AET_av,y_, however can be easily estimated from ET0_av,y_ and R_av,y_ according to a Budyko approach (Budyko, 1974). This assumes that the long-term annual average AET is essentially a property of the climate, and allows its ready calculation as a simple combination of these two predictors. In this work, we compared AET_av,y_ from the daily model simulation with the result of the Budyko model, finding a good correspondence, but with the Budyko model predicting AET_av,y_ on average 39 % higher than the daily step model. After dividing the Budyko model AET_av,y_ by 1.39, the two values can be used interchangeably. Further details are provided in Appendix B.

We tested linear and non-linear univariate regression models by trial and error, based on the shape displayed by the scatterplots of the indicators as a function of each of the predictor variables, and selected the best performing regressions as suggested meta-models, with the help of the correlation matrix depicted in Table 1 to choose the most appropriate predictors (those exhibiting higher R^2^ value).

**Table 1:** Correlation matrix between predictors and indicators.

	*RR/R_av,y_*	Δ*T_ext_*	Δ*T_int_*	*CB*	*ET0_av,y_*	*AET_av,y_*	*AET_av,s_*	*R_av,y_*	*T_med_*	*Rad*
*RR/R_av,y_*	1.00									
Δ*T_ext_*	−0.16	1.00								
Δ*T_int_*	0.57	−0.47	1.00							
*CB*	0.34	−0.05	0.62	1.00						
*ET0_av,y_*	0.36	−0.05	0.75	0.87	1.00					
*AET_av,y_*	−0.25	−0.80	0.12	0.06	−0.01	1.00				
*AET_av,s_*	−0.15	−0.71	−0.19	−0.53	−0.64	0.67	1.00			
*R_av,y_*	−0.80	−0.35	−0.28	−0.13	−0.17	0.73	0.43	1.00		
*T_med_*	0.12	0.38	0.30	0.75	0.79	−0.26	−0.82	−0.17	1.00	
*Rad*	0.30	0.10	0.64	0.86	0.97	−0.08	−0.71	−0.17	0.80	1.00
*Vap*	−0.08	0.42	0.17	0.54	0.65	−0.23	−0.76	−0.04	0.91	0.68

In addition to univariate models, we explored how multivariate linear models could improve the prediction of the indicators. To this end, we tested a few combinations of predictors selected on the basis of correlation with the predictand, and/or the expected relationship among variables based on the physics of the problem, while avoiding use of mutually correlated predictors. The mean average error MAE between the results obtained from the meta-models and those obtained from the full model was calculated as (|X_regr_-X_mod_|)/X_mod_, where X_mod_ is the result calculated applying the full integrated model, while X_regr_ is the value from the regression equation. In an analogous way, the improvements in the accuracy from the monovariate to the multivariate model are calculated as (|X_multiv_-X_monov_|)/X_monov_, where X_monov_ is the MAE or R^2^ of the monovariate model.

## 3 Results

Table 1 shows the correlation matrix of the predictands and predictors. This shows that RR/R_av_,_y_ is well correlated with precipitation R_av,y_ and to a lesser extent with ET0; CB with ET0 and T_med_, while the high correlation with Rad reflects the high correlation between ET0 and Rad; ΔT_ext_ is correlated with AET and to a lesser extent with Vap; ΔT_int_ with ET0 and hence with also Rad. In addition, RR/R_av,y_ shows a closer correlation with R_av,y_ after logarithmic transformation of both variables, and CB and ΔT_int_ show a closer correlation with the logarithm of ET0.

**Fig. 2:**
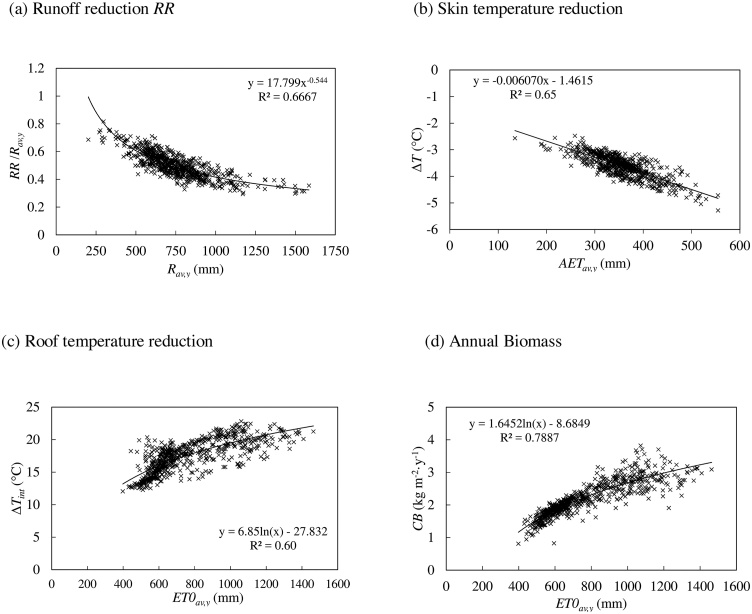
(a) Runoff reduction *RR* normalized to the yearly average rain *R_av,y_* versus *R_av,y_*, for each FUA; (b) surface temperature difference versus yearly averaged value of *AET_av,y_*. Difference of summer temperature between the grey surface and the base temperature of the greened surface versus average annual value of *ET0* (c), and herbaceous biomass versus *ET0* (d).

For each indicator, the best-performing univariate models are shown in Fig. 2 (for a soil thickness of 0.30 m), while those for different soil thickness are reported in Table 2. We use explained variance (R^2^) and mean absolute error (MAE), as performance statistics of the models.

**Table 2:** Coefficients of the empirical equations, obtained from the monovariate analysis at different soil thicknesses. Mean Average Error (MAE) and *R*^2^ are also displayed.

Thickness	Indicator	*R_av,y_*	*AET_av,y_*	*ln ET0_av,y_*	*Interc.*	MAE	*R^2^*
5 cm	*RR/R_av,y_*	13.7 *x*^−0.552^				0.180	0.63
	*ΔT_ext_*		−0.0074		−1.15	0.056	0.66
	*ΔT_int_*			2.56	−9.29	0.067	0.58
	*CB*			0.34	−1.70	0.100	0.67
10 cm	*RR/R_av,y_*	16.4 *x*^−0.556^				0.090	0.66
	*ΔT_ext_*		−0.0070		−1.15	0.058	0.66
	*ΔT_int_*			4.32	−17.50	0.069	0.63
	*CB*			0.69	−3.46	0.096	0.61
20 cm	*RR/R_av,y_*	17.5 *x*^−0.550^				0.087	0.66
	*ΔT_ext_*		−0.0064		−1.34	0.058	0.65
	*ΔT_int_*			6.21	−25.69	0.072	0.62
	*CB*			1.35	−6.79	0.086	0.66
30 cm	*RR/R_av,y_*	17.8 *x*^−0.544^				0.084	0.67
	*ΔT_ext_*		−0.0061		−1.46	0.057	0.65
	*ΔT_int_*			6.85	−27.83	0.075	0.60
	*CB*			1.65	−8.68	0.081	0.79
50 cm	*RR/R_av,y_*	17.2 *x*^−0.528^				0.077	0.67
	*ΔT_ext_*		−0.0060		−1.67	0.071	0.63
	*ΔT_int_*			7.18	−28.48	0.077	0.57
	*CB*			1.98	−10.81	0.080	0.83

The multivariate models, reported in Table 3, were selected by considering the two predictors with highest correlation with each indicator, while avoiding use of mutually correlated predictors (Table 1). Multivariate models, useful for a more accurate prediction, reduce the MAE between 0.2 and 1.3 percentage points, with an R^2^ increase between 2 and 9 percentage points (at 30 cm thickness), with a maximum of MAE improvement of 31 % for the biomass estimation at 10 cm soil thickness.

**Table 3:** Coefficients of the empirical equations, obtained from the multivariate analysis at different soil thicknesses. Mean Average Error (MAE) and *R*^2^ are also displayed.

Thickness	Indicator	*Rad*	*R_av,y_*	*T_med_*	*AET_av,y_*	*ET0_av,y_*	*ln ET0_av,y_*	*Vap*	*Interc.*	MAE	*R^2^*
5 cm	*RR/R_av,y_*		0.00			0.00009			0.302	0.15	0.08
	*ΔT_ext_*				−0.0074			0.00	−1.147	0.056	0.66
	*ΔT_int_*	0.00					2.56		−9.29	0.067	0.58
	*CB*			0.00			0.34		−1.70	0.100	0.67
10 cm	*RR/R_av,y_*		−0.00029			0.000072			0.59	0.086	0.67
	*ΔT_ext_*				−0.0075			0.00	−1.151	0.058	0.66
	*ΔT_int_*	0.00					4.32		−17.50	0.069	0.63
	*CB*			0.047			0.34		−1.63	0.066	0.81
20 cm	*RR/R_av,y_*		−0.00032			0.000083			0.655	0.085	0.68
	*ΔT_ext_*				−0.00587			0.082	−2.405	0.051	0.73
	*ΔT_int_*	−0.077					13.14		−61.22	0.060	0.71
	*CB*			0.017			1.22		−6.13	0.085	0.71
30 cm	*RR/R_av,y_*		−0.00034			0.00009			0.687	0.082	0.69
	*ΔT_ext_*				−0.0056			0.072	−2.386	0.050	0.71
	*ΔT_int_*	−0.091					15.24		−70.19	0.062	0.69
	*CB*			0.031			1.41		−7.45	0.074	0.80
50 cm	*RR/R_av,y_*		−0.00035			0.0001			0.719	0.077	0.71
	*ΔT_ext_*				−0.0057			0.000	−1.665	0.050	0.63
	*ΔT_int_*	0.00					7.18		−28.48	0.077	0.57
	*CB*			0.00			1.98		−10.81	0.079	0.83

The yearly average runoff reduction decreases as the yearly average precipitation increases (Fig. 2a). The term 1-*RR*/*R_av,y_* corresponds to the runoff coefficient of the greened surface, and ranges between 20 % and 60 % as one would expect (Shafique et al., 2018; Soulis et al., 2017; Zhang et al., 2015). A monovariate model in this case would yield *R^2^* = 0.67 and MAE = 8.4 % while the elicited multivariate model for *t =* 0.30 m yields *R*^2^ = 0.69 and MAE = 8% (Table 2, Table 3).

The reduction of land surface temperature ΔT_ext_ shows a good correlation with the yearly average value of the actual evapotranspiration AET_av,y_. The equation that relates ΔT_ext_ to AET_av,y_ is depicted in Fig. 2b (for soil thickness 0.30 m) and in Table 2 for other conditions. The temperature decrease from grey surface to green surface ranges between 2 and 5 °C. Although the R^2^ value is around 0.65, the error percentage between the theoretical value calculated from the daily model and the value estimated by the regression equation is 5.7 %, so that this equation can be used in practical applications. For soil thickness of 0.30 m, the discrepancy between the theoretical value and the value predicted by the multivariate equation one is 5%.

Contrary to Δ*T_ext_*, the difference of temperature at the outer layer of a roof underneath soil, Δ*T_int_*, is not well correlated with *AET*, but it is well explained by *ET0* (Fig. 2c). This occurs because the temperature at the bottom of the soil layer, *T*_base_, remains relatively stable during the year, so that Δ*T_int_* mainly depends on the grey surface temperature *T_grey_*. The latter mainly depends on the climate, of which *ET0*, compounding radiation, humidity, wind intensity and air temperature, is an indicator (the correlation between *T_grey_* and *ET0* exhibits *R*^2^ = 0.90, while between *T_base_* and *ET0*, *R*^2^ = 0.70, and no correlation with *AET*). In the case of thinner soil (*t* =0.05 m), the soil temperature *T_base_* variation during the year is obviously higher; nevertheless, the *ET0* influence on *T_grey_* and *T_base_* remains more significant than the *AET* effect on *T_base_* for *t* =0.05 m, because *AET* reduces with soil thickness (correlation between *T_base_* and *ET0* is *R*^2^ = 0.88 at 5 cm thickness). Considering the soil thickness of 0.30 m, the discrepancy between the theoretical value and the multivariate analysis value is 6.2 % (with *R^2^* higher than 0.69). Overall, temperature differences increase with *Rad*, and this can be seen from the univariate analysis between Δ*T* and *Rad* (Table 1).

Bulk herbaceous biomass production is predicted between 1 and 4 kg/m^2^ per year (10–40 tons per hectare, Fig. 2d). Radiation alone explains 77 % of the variance of yearly biomass production (MAE = 9%), while potential evapotranspiration alone explains 87 % of the variance, with MAE = 8.1 % (see Appendix A for more details).

The meta-models perform evenly across the European region. This can be appreciated from the scatter plots of Fig. 2, showing no outliers nor clustered scatter-points. Likewise, while urban areas have different extents in different regions, the time series that we used to derive our meta-models correspond to areas reasonably uniformly distributed in space, corroborating the generalizability of our results.

Finally, we correlated the equation coefficients of the monovariate meta-models to soil thickness, in order to provide general equations. By plotting these coefficients versus the soil thickness (Table 2) it is possible to see that above 30 cm thickness, the coefficients do not change significantly, or the slope of the monotonic function progressively reduces. Logarithmic equations were selected to interpolate the coefficients (*R*^2^ >0.9), with the exception of the intercept of the meta-model for Δ*T_int_* (which is clearly asymptotic, Eq. 2), obtaining the following equations, that are valid within the range investigated in this work.

(1) Δ*T_ext_* = [0.0007 ln (*t*) -0.0054] *AET* – [0.23 ln (*t*) + 1.7589]

(2) Δ*T_int_* = [2.10 ln (*t*) +9.13] ln (*ET0*) + [19.9 e^−5.86^*^t^* - 30]

(3) *RR*/*R_av,y_* = [1.56 ln (*t*) + 19.267] *R_av,y_*^−0.55^

(4) *CB* = [0.74 ln (*t*) + 2.51] ln (*ET0*) - [4.081 ln (*t*) + 13.475]

The above equations give the following MAE for the 30 cm soil thickness, almost similar to the monovariate models with proper coefficients: 6.3 % for Δ*T_int_* (5.7 % with proper coefficients), 9.5 % for *RR* (8.4 % with proper coefficients), 7.5 % for Δ*T_int_* (7.5 % with proper coefficients), 8.1 % for *CB* (8.1 % with proper coefficients).

Eq. (2) refers to the reduction of roof temperature in summer, with implications on the cooling request in summer. Analogously, green roofs also isolate the building in winter, requiring less heat for a building to be warmed. From our calculations, the average increase of the winter temperature at the external surface of the grey roof (*T_base_*) is 2.1 °C, ranging between 6.3 and -2.4 °C. The negative values mean that the temperature in winter is lower with the green roof, thus that the green roof does not reduce the heating request. However, the negative values only occur in 73 FUAs out of 671, and typically in FUAs characterized by high *ET0* and Radiation, where in winter the frequent sunny days can warm the grey roof surface, thus reducing the heating request. In these areas we expect the benefits from a reduction of the summer temperature to outweigh the disadvantages from a reduction of the temperature in winter, globally justifying the implementation of a green roof for energy purposes.

## 4 Discussion

In their exploratory intent, our calculations are referred to hypothetical situations, and as such they cannot be validated. However, the underlying equations are extensively applied and generally considered realistic, especially in the European context. Moreover, we benchmarked our results against the available literature as further discussed below. The equations of the SWAT model, as used in our analysis, have been applied in Malagó et al. (2007) for the Danube basin, in Samadi (2017) for the United States, and in Wang et al. (2018) for the Tennessee river basin, while the hydrological model of Pistocchi et al. (2008) has been tested against data in various cases across Europe (Pistocchi et al., 2008; Santi et al., 2013) and proven realistic.

While the regression equations cannot be validated in the stricter sense, we nonetheless checked that the variables predicted by the model were in line with available evidence. Our calculated difference between heat flux to the roof in case of grey surface and heat flux to the soil surface in case of green surface varies between 88 and 173 W/m^2^, coherently with La Roche and Berardi (2014) and Sun et al. (2013), where differences between 30 and 340 W/m^2^ were recorded. Issa et al. (2015) found that the average decrease in the roof inner temperature (at 0.07 m depth) between the green and control roofs ranged from 4 to 7 °C, while in our calculation the decrease was between 5 and 9 °C for a 0.05 m thick soil. In Xiao et al. (2018), a validated estimation of the effect of vegetation density (*P*), expressed in percentage (thus *P* ranged between 0 and 100) on the reduction of surface temperature *T_skin_* in summer, is presented for Vienna and Madrid. Temperature is reduced by 3 °C for Vienna, and 3.75 °C for Madrid, as a consequence of the greening of a grey urban surface. Similar results were obtained by applying our theoretical model: a reduction of 3.8 °C for Vienna and 3.4 °C for Madrid, using a herbaceous crop and a soil 0.30 m thick. Overall, these comparisons show that our model is realistic.

Our predicted runoff reduction (between 40 % and 80 % for *t* =30 cm) compares favorably with Soulis et al. (2017) predicting 17–100 % of runoff reduction for a soil 8 cm thick, and 26–83 % with our model; Schultz et al. (2018) found an average *RR* of 33 % for a 12.5 cm thick soil, while our reduction with 12.5 cm thickness ranged between 27 % and 87 %, and Zhang et al. (2015) found a water retention between 35%–100% with 15 cm soil thickness (28 % and 88 % with our model); Shafique et al. (2018) calculated a *RR* between 10 % and 60 % for a soil thickness of 3 cm, comparable with our retention between 24 % and 75 % for a soil thickness of 5 cm. Results are also in agreement with Johannessen et al. (2017), where the warmest and driest locations (lower annual precipitation) showed highest retention in percentage of annual precipitation.

## 5 Conclusions

In this contribution we illustrate a method to quantify the beneficial effects of urban greening in terms of runoff reduction, biomass growth and surface temperature reduction indicators, developing a satisfactory surrogate to a complex model. We show that our simple equations, relating these indicators to easily accessible environmental variables, are consistent with the results one could obtain by applying a complex, integrated model with standard parameters. The discrepancy compared to more complex model results are below 9% for runoff, below 9% for biomass, and below 6% for surface temperature. Therefore, the meta-models proposed in this paper can be applied to estimate urban greening effects in the European context as a first approximation. The proposed equations are empirical, hence their validity outside of the range of conditions investigated in this study is questionable. However, the ample range of latitudes from 35° (Cyprus) to 65° (Finland), and of elevation from below 0 m (the Netherlands) to above 1600 m (Austria) make them arguably suited for applications across all temperate regions.

The proposed metamodels provide a quick tool for the appraisal of urban development and urban greening strategies. Water retention by urban green infrastructure may contribute to reducing floods and pollution. Biomass growth may contribute to carbon sequestration and may yield economically useful production for people (berry crops) or livestock (grass); it can also encourage social activities like gardening. The reduction of temperature at the bottom of a soil layer on urban surfaces is directly proportional to the cost saving by reduced energy needs for summer cooling, if we assume that the building internal volume under the roof is to be cooled. The reduction of surface temperatures associated to greening yields also a potential benefit in terms of heat islands mitigation. The proposed equations enable a reasonably accurate quantification of all these benefits at a first level of approximation, in order to support the planning of resilient cities against the effects of climate changes. Quaranta et al. (2021), present an application of the metamodels for continental scale assessment of urban greening.

## Author statement

AP and EQ conceived the research; CD curated the data; EQ developed the calculations under the supervision of AP; EQ and AP wrote the manuscript.

## Declaration of Competing Interest

Authors declare that they do not have conflict of interest.

## Figures and Tables

**Table A1:** Results of the sensitivity analysis. The reference condition is that of alfalfa (parameters: *t* =0.30 m, Soil Class 3, LEC = 0.3, RUE = 2). Each percentage expresses the discrepancy between the modified condition (by varying one parameter at time and keeping the other constant) and the reference condition.

	*t* =0.05 m	*t* =0.50 m	Class 1	Class 5	LEC = 0.1	LEC = 1	RUE = 1	RUE = 4
Runoff	35.7 %	−8.8%	12.2 %	−20.7%	2.0 %	0.3 %	2.8 %	0.8 %
*T_base_*	81.3 %	−13.0%	−1.8%	1.3 %	0.2 %	−0.2%	0.2 %	−0.4%

**Fig. A1:**
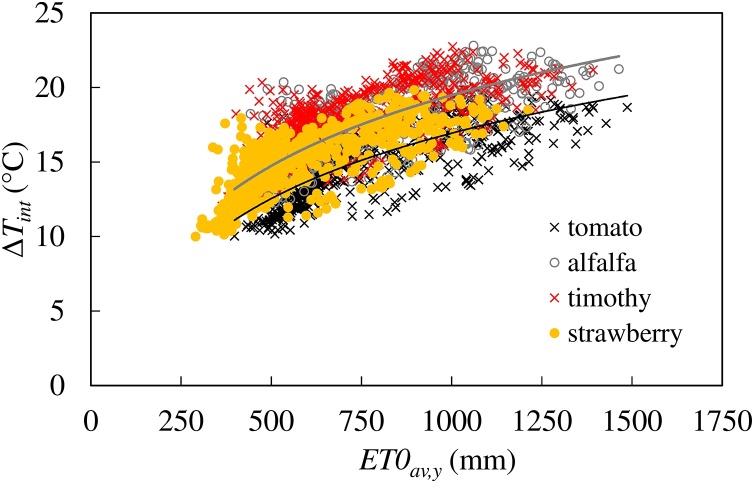
Reduction of roof temperature versus evapotranspiration. Comparisons among different crops.

**Fig. A2:**
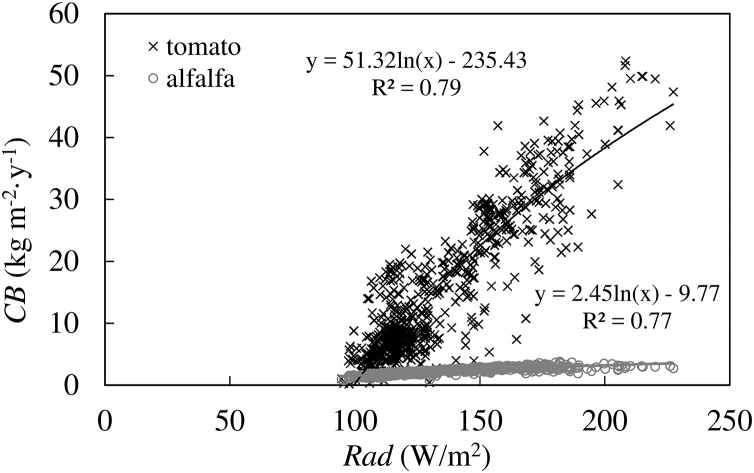
Bulk biomass of tomato and alfalfa versus radiation.

**Fig. A3:**
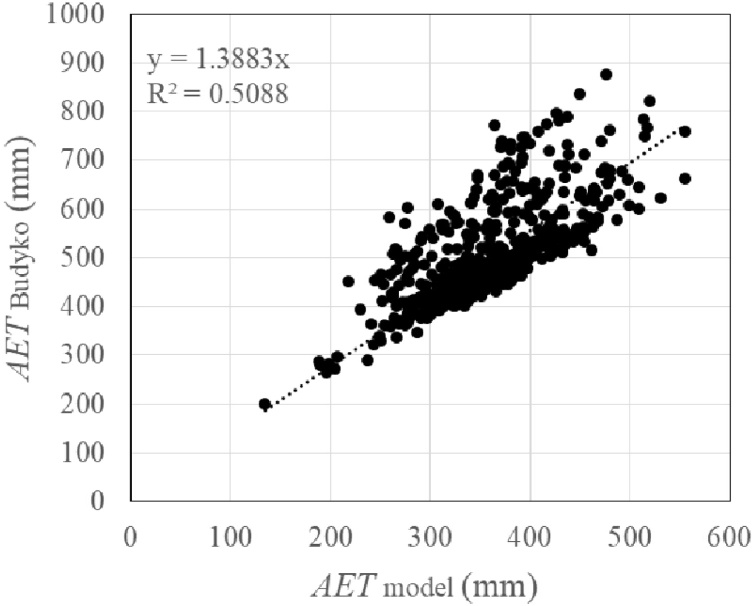
*AET* estimated by Budyko is 39 % higher than the calculated (and more accurate) *AET*.

## Appendix A

The choice of the herbaceous crop type is not an essential parameter, so that large scale analysis can be performed almost neglecting the herbaceous type. This is confirmed in Table A1, where it can be seen that the effect of LEC and RUE is negligible in comparison with the effects of soil thickness, that was indeed here also investigated.

In Fig. A1 alfalfa, selected as reference crop, was compared with other herbaceous and berry plant to prove the minimal effect of vegetation type on the results. In Fig. A1 the reduction of the roof temperature is shown versus *ET0* for alfalfa, tomato, timothy and strawberry (parameters based on SWAT database, and on Heuvelink, 1996; Nangare et al., 2016; Olson, 1981; Pathak and Stoddard, 2018). The reduction of the temperature induced by tomato is lower than that induced by the herbaceous plants, due to the lower *AET* of berry plants (a portion of water is stored inside the fruit). However, results of berry crops and that of herbaceous crops are almost similar.

Fig. A2 also depicts the wet biomass of tomato and alfalfa versus the radiation (Fig. 2d already shows the herbaceous *CB* versus *ET0*), and some discussions are provided on the robustness of the estimation. Bulk herbaceous biomass production is predicted between 1–4 kg/m^2^ per year (10–40 tons per hectare), while the berry production between 0.2 and 50 kg/m^2^ (including the weight of plants and stems). It is worth noting that *CB* refers to the bulk biomass of the whole plant: in the case of tomato, the weight of fruits alone is slightly less than one half of the total plant weight (Heuvelink, 1996). Radiation alone explains 77 % of the variance of yearly biomass production (MAE = 9%), while potential evapotranspiration alone explains 79 % of the variance, with MAE = 8.1 % for alfalfa. For tomato, radiation explains 78 % of the variance, with MAE = 53 %. When considering the biomass of tomato, the highest MAE are those whose *CB* value (calculated by the integrated model) is below 7 kg/m^2^; if results below *CB* =7 kg/m^2^ were discarded, MAE reduces from 38 % to 17 %.

Tomato results are in agreement with Nangare et al. (2016), where the *Lycopersicon esculentum Mill* was cultivated at an average temperature of 22 °C, with an average yield of 7 kg/m^2^ of fruits. By our model, considering that temperature, the total biomass would be 24 kg/m^2^, that corresponds to about 10 kg/m^2^ of fruits. Kuscu et al. (2014) measured a fruit yield of 32 tons/ha at 14 °C of average temperature, i.e. 3.2 kg/m^2^, that corresponds to 6.5 kg/m^2^ of total plant yield. Our estimation gives a total of 2.3 kg/m^2^. Although in this case the discrepancy is higher, it must be noted that this yield example is among the less productive ones, thus a case that is more difficult to be predicted; anyway, the order of magnitude, which is of our interest in this study, is captured.

## Appendix B

Budyko (1974) assumes that the long-term *AET* is essentially a property of the climate, hence, for our case, this allows its readily calculation as a simple combination of the annual average *ET0*_av,y_ and *R*_av,y_. We compared *AET*_av,y_ from the daily model simulation with the result of the Budyko model (Eq. A1), finding a good correspondence, but with the Budyko model predicting *AET*_av,y_ on average 39 % higher than the daily step model. After dividing the Budyko model *AET*_av,y_ by 1.39, the two values can be used interchangeably (Fig. A3).

(A1) $$\frac{\text{AET}}{\text{R}}\text{= }\sqrt{\frac{\text{ET0}}{\text{R}}\text{ tanh}\frac{\text{R}}{\text{ET0}}\;\left(\text{1-}{\text{e}}^{-\frac{\text{ET0}}{\text{R}}}\right)}$$
